# Augmentation of hepatoprotective potential of *Aegle marmelos* in combination with piperine in carbon tetrachloride model in wistar rats

**DOI:** 10.1186/s13065-018-0463-9

**Published:** 2018-08-20

**Authors:** Deepti Rathee, Anjoo Kamboj, Shabir Sidhu

**Affiliations:** 1 Department of RIC, IKG Punjab Technical University, Kapurthala, Punjab India; 2 Chandigarh College of Pharmacy, Chandigarh Group of Colleges, Landran, Punjab India; 3 Department of Food Science and Technology, I. K. Gujral Punjab Technical University, Main Campus, Kapurthala, Punjab 144603 India

**Keywords:** *Aegle marmelos*, Rutin, Silymarin, HPLC, Anti-inflammatory, IL-10 and TNF-α levels, Oxidative stress

## Abstract

The current study investigated hepatoprotective and antioxidant effects of *Aegle marmelos* leaves extract. The major constituent present in the extract i.e. rutin was quantified by using HPLC. Further, the study explored hepatoprotective effect of *A. marmelos* (70% ethanol extract) in combination with piperine. The normal control and carbon tetrachloride (CCl_4_) administered rats were divided into 7 groups. Hepatic damage biomarkers were determined in serum samples and oxidative stress biomarkers (malondialdehyde, reduced glutathione, glutathione reductase, glutathione peroxidase, glutathione-S-transferase, superoxide dismutase and catalase), pro-inflammatory and anti-inflammatory cytokines were determined in liver homogenates. CCl_4_ caused marked liver damage as evident by significant increased activities of serum alkaline phosphatase, bilirubin, lactate dehydrogenase, alanine aminotransferase, aspartate aminotransferase, Interleukin 10 and Tumor necrosis factor-α levels compared to normal control. The oxidative stress parameters also significantly modulated in CCl_4_ group as compared to normal control. Treatment with *A. marmelos* reduced the severity of toxicity in a dose dependent fashion and the results of *A. marmelos* extract 50 mg/kg group were comparable to silymarin group. The low dose of *A. marmelos* extract (25 mg/kg) per se did not significantly reversed the hepatotoxicity but low dose of *A. marmelos* in combination with piperine showed significant reversal of hepatotoxicity. In conclusion, *A. marmelos* exerts potential hepatoprotective activity through its antioxidant and anti-inflammatory properties which was enhanced by co-treatment with piperine.
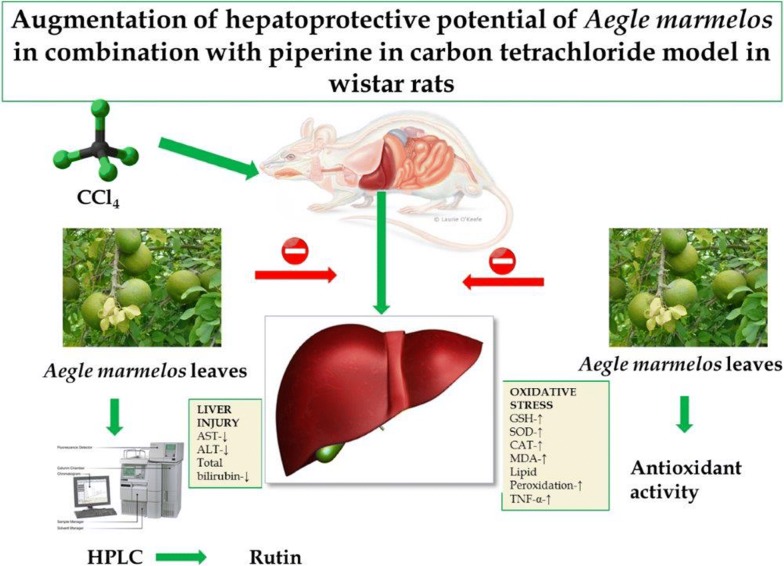

## Introduction

*Aegle marmelos*, commonly known as Bael, a spiny tree of Rutaceae family is an indigenous tree found in India, Myanmar, Pakistan and Bangladesh. The leaves, roots, bark, seeds and fruits are edible and have medicinal values. The root is an important ingredient of the ‘Dasmula’ (ten roots) recipe [1]. Ayurveda describes the medicinal properties of this plant. Ayurvedic literature claims various pharmacological properties of Bael leaves. Activities include astringent, laxative, and expectorant, useful in treatment of ophthalmia, deafness, inflammations, cataract, diabetes, diarrhoea, dysentery, heart palpitation, and asthmatic complications [2]. Increased use of *A. marmelos* as a medicinal agent in different systems of medicine including folk medicine, various research studies undertaken in recent past to explore the therapeutic potential of different parts of the plant. A number of studies showed antifungal [3], ulcer healing [4], anti-inflammatory [5] and anti-diabetic [6] properties of *A. marmelos*. Literature also reports diuretic [7], anti-fertility [8], hepatoprotective activities [9] and anticancer properties [10].

Economics of treatment, linked to drug dosage, has led to new drug development strategies. Piperine is an amide alkaloid found in the fruits of black and long pepper plants (*Piper nigrum Linn* and *Piper longum Linn*). Black pepper has several uses in Ayurvedic medicine, the effects of which are attributed to piperine. Piperine is reported to have many pharmacological activities such as analgesic and anti-inflammatory [11] and usefulness in various gastrointestinal disorders [12]. Hepatoprotective activity of piperine has also been reported [13].

CCl_4_ is a widely known experimental hepatotoxin. It accumulates in hepatic parenchymal cells and metabolically activated by cytochrome P-450 dependent monoxygenases forming a trichloromethyl free radical (CCl_3_). This free radical alkylates cellular proteins and other macromolecules with a simultaneous attack on polyunsaturated fatty acids in the presence of oxygen to produce lipid peroxides. This causes alterations in the Ca^++^ homeostasis resulting in cell death [14]. The effects of CCl_4_ on hepatocytes are manifested histologically as hepatic steatosis (e.g. fatty infiltration), centrilobular necrosis and cirrhosis depending upon dose and exposure time. Hepatic steatosis of the liver is a multifactorial phenomenon and is thought to occur due to blockage of lipoprotein secretion [15], impaired synthesis or peroxidation of phospholipids, or both [16]. Considering the diverse medicinal properties of *A. marmelos*, the present study explored protective effects of *A. marmelos* leaves and the effect of co-administration of piperine against CCl_4_ induced hepatotoxicity in rats.

## Materials and methods

### Chemicals and instruments

All the chemicals were purchased from Thermo Fisher Scientific. High performance liquid chromatography (HPLC) was performed on Agilent technologies HPLC system with column from Agilent eclipse XBD^®^; Serum biomarkers were used as Accurex kits (Accurex Biomedical Pvt. Ltd, India); Graph Pad Prism (Version 5) from San Diego, CA, USA; Piperine and Silymarin from Sigma-Aldrich, USA.

### Collection, authentication and extraction of *A. marmelos* leaves

Collection of *A. marmelos* leaves was undertaken from areas in and around Chandigarh, India during the month of January. Dr. Sujata Bhattacharya, Assistant Professor, School of Biological and Environmental Sciences, Shoolini University, Solan authenticated the plant material. Voucher specimens of the plant (SUBMS/89) were deposited in the School of Biological and Environmental Sciences, Shoolini University, Solan.

The dried coarsely powdered leaves of the plant (500 g) were first extracted with the petroleum ether followed by 70% ethanol by the hot extraction process using a Soxhlet apparatus [17, 18]. The solvent removed by distillation under reduced pressure after completion of extraction process and the prepared extract was stored in vacuum desiccator until further use.

### Phytochemical screening of *A. marmelos* leaves hydro-alcoholic extract

#### Preliminary phytochemical screening

The extract was tested for the presence of bioactive compounds by using the standard methods explained by previously [17, 18]. Preliminary phytochemical screening was carried out to confirm the presence of alkaloids, carbohydrates, flavonoids, fixed oils and fats, tannins and phenolic compounds, phytosterols, protein/amino acids and saponins by using standard procedures described by Harborne [17] and Kokate [18].

### Quantitative determination of Rutin

The rutin content of the extract was determined chromatographically using HPLC system [19, 20] of Agilent technologies, with column from Agilent eclipse XBD^®^ C 18 bonded with 5 µm (4.6 × 150 mm). Before starting validation, system suitability parameter was calculated. It was determined by taking percent relative standard deviation (RSD) of the five standards injections using the same concentration of rutin by HPLC method. The precision of system was checked as per the developed method by using multiple injections of a homogeneous standard solution. This indicated the performance of the HPLC instrument under the chromatographic condition. As a part of method validation minimum five injections of the standard preparation were performed for inter day precision. The relative standard deviation was not more than 2.0%. Limits of detection (LOD) and Limit of quantification (LOQ) were calculated by method based on standard deviation (σ) and slope (S) of calibration plot using formula LOD = 3.3 σ/S and LOQ = 10 σ/S.

### In vitro antioxidant study of *A. marmelos leaves* extract

The DPPH or 2,2-diphenyl-1-picrylhydrazyl assay was performed using the method of Molyneux [21]. Then the absorbance recorded at 515 nm. The standard curve was linear between 25 and 800 mM Trolox. Results are expressed in mMTE/g fresh mass. ABTS or 2,2′-azino-bis(3-ethylbenzothiazoline-6-sulphonic acid) assay was also used to evaluate antioxidant potential of the extract [22]. Results are expressed by comparison with standard amounts of the synthetic antioxidant trolox (a water-soluble vitamin E analogue) to give rise to the Trolox equivalent antioxidant capacity (TEAC). The total antioxidant activity of *A. marmelos* was evaluated by Ferric reducing ability of plasma (FRAP) method [23]. The results were expressed as ascorbic acid equivalent antioxidant capacity (AEAC).

### Experimental protocol

#### Animal husbandry

Wistar albino rats (either sex) 4–6 months of age weighing 180–200 g were supplied by Chandigarh college of Pharmacy, Landran (Punjab, India). The rats were housed in a temperature-controlled (25 ± 1 °C) environment and provided free access to pellet food and purified drinking water. Animals were acclimatized to laboratory conditions 1 week prior to start of experiments. All animal experiments performed in accordance with the guidelines of Committee for the Purpose of Control and Supervision of Experiments on Animals (CPCSEA), Government of India, between 08:00 h and 14:00 h. The Institutional Animal Ethics Committee (IAEC) approved the animal experimentation protocols (1201/a/08/CPCSEA). Rats were randomly divided into seven groups of six animals each:

i. Normal control; animals of this group were fed pellets and water ad libitum for 15 days.
ii. Drug control; rats were administered 50 mg/kg body weight leaf extract of *A. marmelos* for 15 days.
iii. CCl_4_ group; rats were administered only 3 ml/kg CCl_4._
iv. Positive control; rats were administered CCl_4_ + 200 mg/kg silymarin.
v. *A. marmelos* extract 25 group; rats were administered CCl_4_ +* A. marmelos* extract 25 mg/kg.
vi. *A. marmelos* extract 50 group; rats were administered CCl_4_ +* A. marmelos* extract 50 mg/kg.
vii. Piperine group; rats were administered CCl_4_ +* A. marmelos* extract 25 mg/kg + piperine 20 mg/kg.

All the drugs were administered orally for 15 days; CCl_4_ was administered once on the fifth day of the treatment period in a dose of 3 ml/kg body weight intraperitoneal (i.p.) [24]. The dose of *A. marmelos* used in the present study was based upon the lethal dose (LD_50_) values [25]. The doses of silymarin 200 mg/kg [26] and piperine at 20 mg/kg [27] were selected from literature reports.

The animals were fasted overnight before sacrificing. On the day of sacrifice rats received their respective drugs and 2 h later were injected with thiopentone (50 mg/kg i.p.), and blood was withdrawn by cardiac puncture. The blood was centrifuged at 4000*g* for 15 min at 4 °C and serum separated. The liver was removed and washed in ice-cold saline solution. A part of it homogenized in phosphate buffer saline (0.1 M PBS, pH 7.4). The homogenates centrifuged at 4000*g* for 20 min at 4 ^ο^C and supernatant was stored at − 80 °C.

#### Hepatic damage serum biomarkers

Hepatic damage serum biomarkers, alkaline phosphatase (ALP), bilirubin, lactate dehydrogenase (LDH), alanine aminotransferase (ALT) and aspartate aminotransferase (AST) were measured by an auto-analyzer using the Accurex kits (Accurex Biomedical Pvt. Ltd, India). The total protein was estimated by Lowry [28] method.

#### Oxidative stress parameters

Evaluation of oxidative stress parameters was done in liver homogenates. Malondialdehyde (MDA) level in the liver was determined according to the method of Ohkawa [29]. Results are expressed as nM MDA/mg of protein. Reduced glutathione level was estimated by the method of Ellman [30]. The results are expressed as μg/mg of protein. Superoxide dismutase (SOD) activity was estimated according to method of Robak [31]. The results are expressed as U/mg of protein. Catalase (CAT) activity was measured by the method of Aebi [32]. The results are expressed as µM of hydrogen peroxide decomposed/mg of protein. Glutathione reductase (GR) activity was measured by the method of Carlberg [33]. The rate of Nicotinamide adenine dinucleotide phosphate (NADPH) oxidation is directly proportional to the GR activity in the sample. GR activity is expressed as nM of NADPH oxidized/min/mg of protein. GSH-S-transferase (GST) activity was measured spectrophotometrically by the method of Habig [34]. GST enzyme activity was calculated as nM of CDNB–GSH conjugate formed/min/mg of protein. Glutathione peroxidase (GPx) activity was calculated as described by Athar [35]. The activity was recorded at 340 nm and expressed as nM of NADPH oxidized/min/mg of protein. Glucose-6-phosphate dehydrogenase (G6PD) activity was determined by the method of Zaheer [36]. The changes in absorbance were recorded at 340 nm and enzyme activity was calculated as nM of NADPH formed/min/mg of protein. The total protein was estimated by Lowry method [28].

#### Inflammatory markers (IL-10 and TNF-α level)

IL-10 and TNF-α level in serum were estimated by Enzyme-Linked Immunosorbent Assay (ELISA) method. The concentration of the cytokines in 100 µl sample volume was determined according to the manufacturer’s protocol. IL-10 and TNF-α concentrations are expressed as pg/ml.

#### Histopathological examination

Liver of rats from different groups was fixed in 10% neutral buffered formalin. After fixation, liver samples were dehydrated in alcohol, cleared in xylene, and embedded in paraffin wax 56 °C in hot air oven for 24 h. Paraffin embedded tissue blocks were prepared for sectioning at 5 mm thickness by a micro-tome. The obtained tissue sections were collected on glass slides, deparaffinized, and stained by hematoxylin and eosin (H&E) stain for histopathological examination through the light microscope.

### Statistical analysis

Results expressed as mean ± SEM (standard error mean). The statistical analysis was done using program Graph Pad Prism 5.0 Version for Windows (San Diego, CA, USA). The data were analyzed statistically by using one way analysis of variance (ANOVA). In case ANOVA showed significant difference, post hoc analysis performed with Tukey’s test. *p *< 0.05 was considered to be statistically significant.

## Results

### Collection, authentication and extraction of *A. marmelos* leaves

The collected leaves were authenticated and the hydro-alcoholic extract was prepared and stored in desiccator for further use. The percentage yield obtained was 18.2% w/w.

### Phytochemical screening of *A. marmelos* leaves extract

Results of the preliminary phytochemical screening of leaves extract showed the occurrence of alkaloids, carbohydrates, flavonoids, tannins, phenolic compounds and phytosterols (Table 1). Literature reports that the hydro-alcoholic leaf extract of *A. marmelos* has maximum amount of flavonoid and phenolic compounds, rutin being the major component [37, 38]. The presence of the polyphenolic compound rutin was further confirmed by HPLC studies.

**Table 1:** Qualitative chemical tests of the extracts of *A. marmelos* leaves

Class of compound	Petroleum ether	Hydroalcoholic
Alkaloids		
Dragendorff’s test	−	+
Mayer’s test	−	+
Hager’s test	−	+
Wagner’s test	−	+
Carbohydrates		
Molish’s test	−	+
Fehling’s test	−	+
Benedict’s test	−	+
Flavonoids		
Shinoda test	−	+
Lead acetate test	−	+
Fixed oils and fats		
Spot test	−	−
Saponification test	−	−
Tannins and phenolic compounds		
5% FeCl_3_ solution	−	+
Lead acetate solution	−	+
KMnO_4_	−	+
K_2_Cr_2_O_7_	−	+
Gelatin solution	−	+
Phytosterols		
Salkowiski test	+	−
Liebermann–Burchard test	+	−
Proteins and amino acids		
Biuret test	−	−
Million’s test	−	−
Ninhydrin test	−	−
Saponins		
Foam test	−	−

### HPLC analysis

**Fig. 1:**
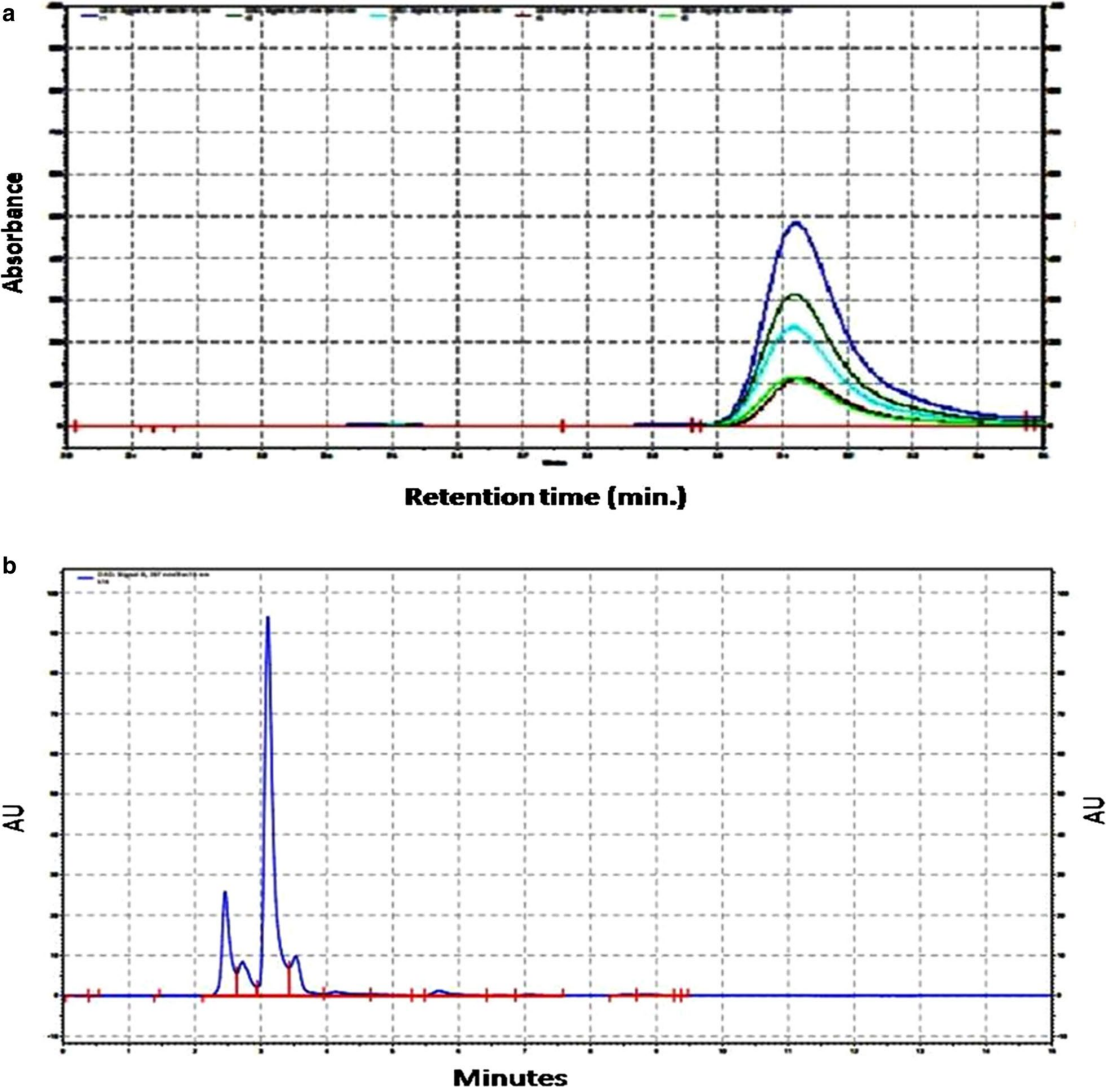
**a** Overlay chromatogram at 257 nm at different concentrations. **b** Typical chromatogram with optimized conditions gave sharp and symmetric peak with specific retention time of rutin in the leaves of *A. marmelos*

Validation and optimization of chromatographic conditions for reverse phase HPLC (RP-HPLC) method for estimation of rutin was performed (Table 2). The mobile phase combination of methanol, acetonitrile and water in the ratio of 40:15:45 containing 1.0% acetic acid v/v, injection volume 10 µl and flow rate of 1 ml/min using UV detector at 257 nm was used. An overlay chromatogram was prepared at 257 nm with different concentrations (Fig. 1a). A calibration curve of rutin was prepared using different concentrations (100, 200, 300, 400 and 500 µg/ml) of pure rutin. Typical chromatogram with optimized condition gave sharp and symmetric peak with specific retention time of 3.107 ± 0.0145 min (Fig. 1b). The percent relative intraday standard deviation (%RSD) values were 0.342–0.786 μg/ml and those for inter day precision were 0.411–0.547 μg/ml respectively. The derived LOD and LOQ for rutin were determined to be 0.6 and 1.7 μg/ml, respectively.

**Table 2:** Validation and optimization of HPLC

Sr. no.	Validation parameter	Results	
1	Linearity range	5–25 ppm	
2	Regression equation	Slope	36081
		Intercept	351181
3	Regression coefficient	0.9	
4	Precision	%RSD first day	0.6
		%RSD second day	0.1
5	Accuracy (% recovery)	SD	1.2
		%RSD	1.2
		Mean	100.7
6	LOD (μg/ml)	0.6	
7	LOQ (μg/ml)	1.7

### Antioxidant activity

The three assays demonstrated the potential antioxidant ability of leaf extract of *A. marmelos* (Table 3). The extract exhibited concentration dependent ability to quench DPPH free radical. In this assay, 85.3 ± 2.2% inhibition was achieved at concentration 640 μg/ml. The IC_50_ of extract was 160.9 μg/ml and that of Trolox was 9.2 μg/ml. The extract also showed significant ABTS scavenging potential, 640 μg/ml contraction showed 90% inhibition. The IC_50_ of extract in ABTS assay was 134.54.2 μg/ml and for Trolox it was 4.2 μg/ml. Further to this, the extract also showed an ability to donate electrons to convert Fe^3+^→Fe^2+^ as indicated by the concentration dependent increase in the percentage reducing power. However, the maximum inhibition (at 640 μg/ml) observed significantly lower compared to DPPH and ABTS assay. The IC_50_ in FRAP assay was observed at 424.5 μg/ml which is significantly higher than the other two assay.

**Table 3:** Antioxidant potential of *A. marmelos* leaves extract

*A. marmelos* extract conc. (μg/ml)	Percentage inhibition		
	DPPH assay	ABTS assay	FRPS assay
10	8.8 ± 1.0	3.1 ± 1.1	3.8 ± 0.7
20	13.6 ± 1.6	7.4 ± 1.2	7.3 ± 0.7
40	22.7 ± 1.9	15.2 ± 2.3	13.5 ± 0.9
80	36.0 ± 1.7	34.5 ± 2.0	22.1 ± 1.7
160	49.0 ± 2.5	56.0 ± 3.9	31.5 ± 1.5
320	75.4 ± 3.4	73.4 ± 2.8	42.2 ± 2.6
640	85.3 ± 2.2	90.0 ± 1.5	53.0 ± 2.7
Extract IC_50_	160.9	134.5	424.5
Trolox IC_50_	9.2	4.2	–

Data presents as mean ± SEM (*n* = 5)

### *A. marmelos* treatment and serum biochemical parameters

**Fig. 2:**
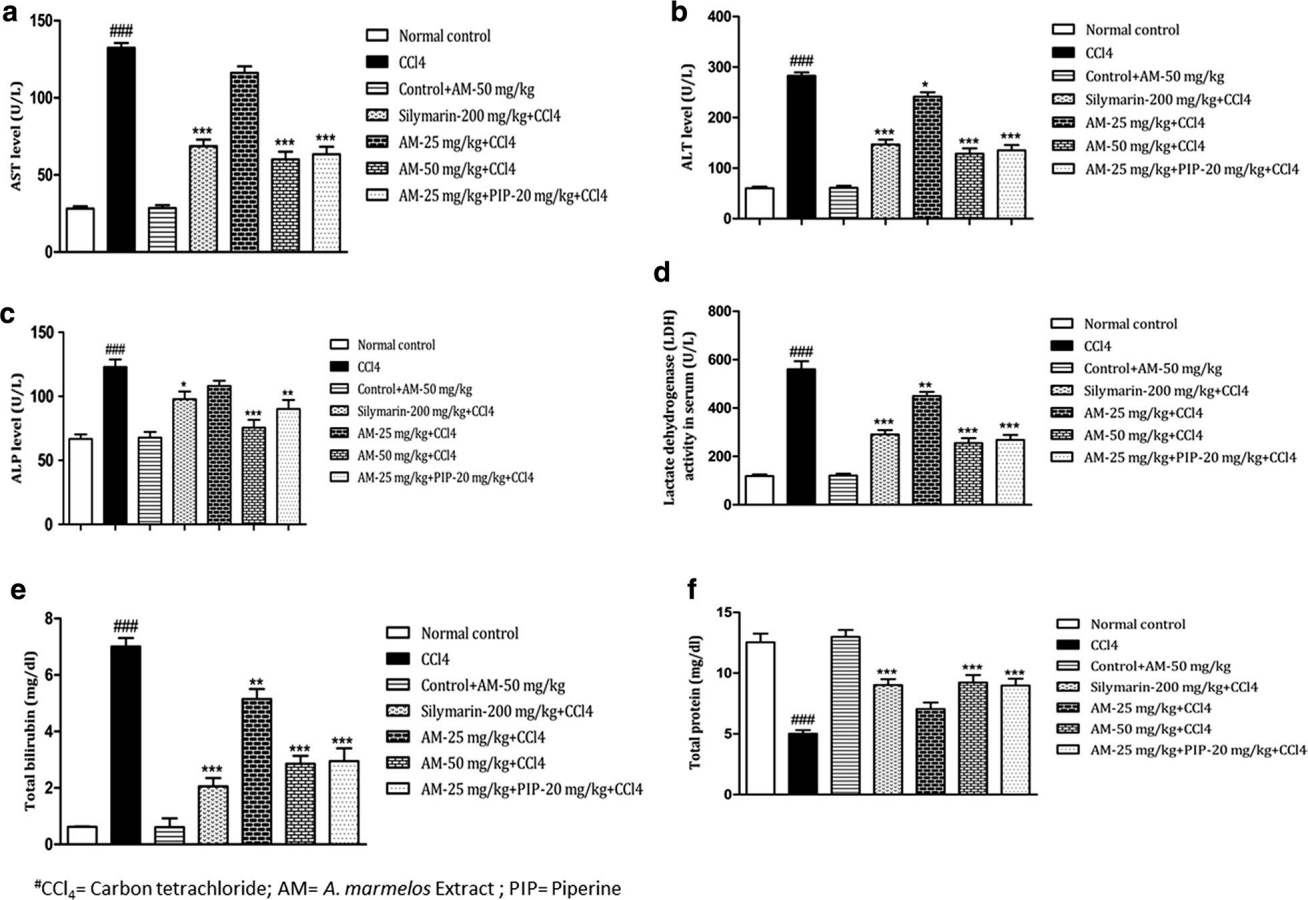
Effect of *A. marmelos* extract on **a** aspartate aminotransferase (AST); **b** alanine aminotransferase (ALT); **c** alkaline phosphatase (ALP); **d** lactate dehydrogenase (LDH); **e** total bilirubin; **f** total protein in carbon tetrachloride (CCl_4_) induced hepatotoxicity in rats (data presented mean ± SEM; (n = 6); *p < 0.05; **p < 0.01; ***, ^###^p < 0.001; ^#^ vs Control; * vs CCl_4_)

CCl_4_ administration resulted in significant liver damage as revealed by the elevated level of serum hepatic enzymes (AST, ALT, ALP and LDH) and reduced level of protein and increased level of total bilirubin. CCl_4_ treated group showed significant increase in AST (*p *< 0.001) and ALT (*p *< 0.001) levels as compared to normal control group. Treatment with *A. marmelos* (50 mg/kg) significantly (*p *< 0.001) reduced AST and ALT levels as compared to CCl_4_ group. Moreover, co-administration of *A. marmelos* 25 mg/kg and piperine significantly (*p *< 0.001) lowered the AST and ALT level as compared to CCl_4_ group (Fig. 2a, b). Whereas, *A. marmelos* 25 mg/kg failed to lower the elevated levels significantly. The results of *A. marmelos* extract 50 group and *A. marmelos* extract 25 + Piperine group were comparable to that of silymarin group.

When compared with normal control group, ALP and LDH levels were found to be significant (*p *< 0.001) higher in CCl_4_ treated group. *A. marmelos* 50 mg/kg and *A. marmelos* 25 mg/kg + piperine treatments significantly ameliorated the elevated ALP level (*p *< 0.001 and *p *< 0.01 respectively) as compared to CCl_4_ treated group (Fig. 2c). The *A. marmelos* extract 25 mg/kg treatment lowered the elevated levels but not significantly as compared to CCl_4_ group. LDH level was significantly lowered by the treatment with *A. marmelos* 25 mg/kg (*p *< 0.01), *A. marmelos* 50 mg/kg (*p *< 0.001) and *A. marmelos* 25 mg/kg + piperine (*p *< 0.001) (Fig. 2d). The ALP and LDH levels in *A. marmelos* 50 mg/kg group and *A. marmelos* 25 mg/kg + piperine groups were comparable to silymarin group with no significant difference. Furthermore, administration of CCl_4_ significantly reduced the total protein level (*p *< 0.001) and increased the total bilirubin level (*p *< 0.001) as compared to normal control group. Total bilirubin level was dose dependently reduced with *A. marmelos* treatment (Fig. 2e). *A. marmelos* 25 mg/kg (*p *< 0.01), *A. marmelos* 50 mg/kg (*p *< 0.001). Total bilirubin level in *A. marmelos* extract 25 mg/kg + piperine group was significantly lower as compared to *A. marmelos* extract 25 mg/kg group. Total protein was significantly increased with *A. marmelos* extract 50 mg/kg (*p *< 0.001) treatment and *A. marmelos* 25 mg/kg + piperine (*p *< 0.001) as compared to CCl_4_ group (Fig. 2f). The total protein and bilirubin levels in *A. marmelos* 50 mg/kg and *A. marmelos* extract 25 mg/kg + piperine group were comparable to silymarin group. The results of drug control group were comparable to normal control group.

### *A. marmelos* treatment and oxidative stress

**Fig. 3:**
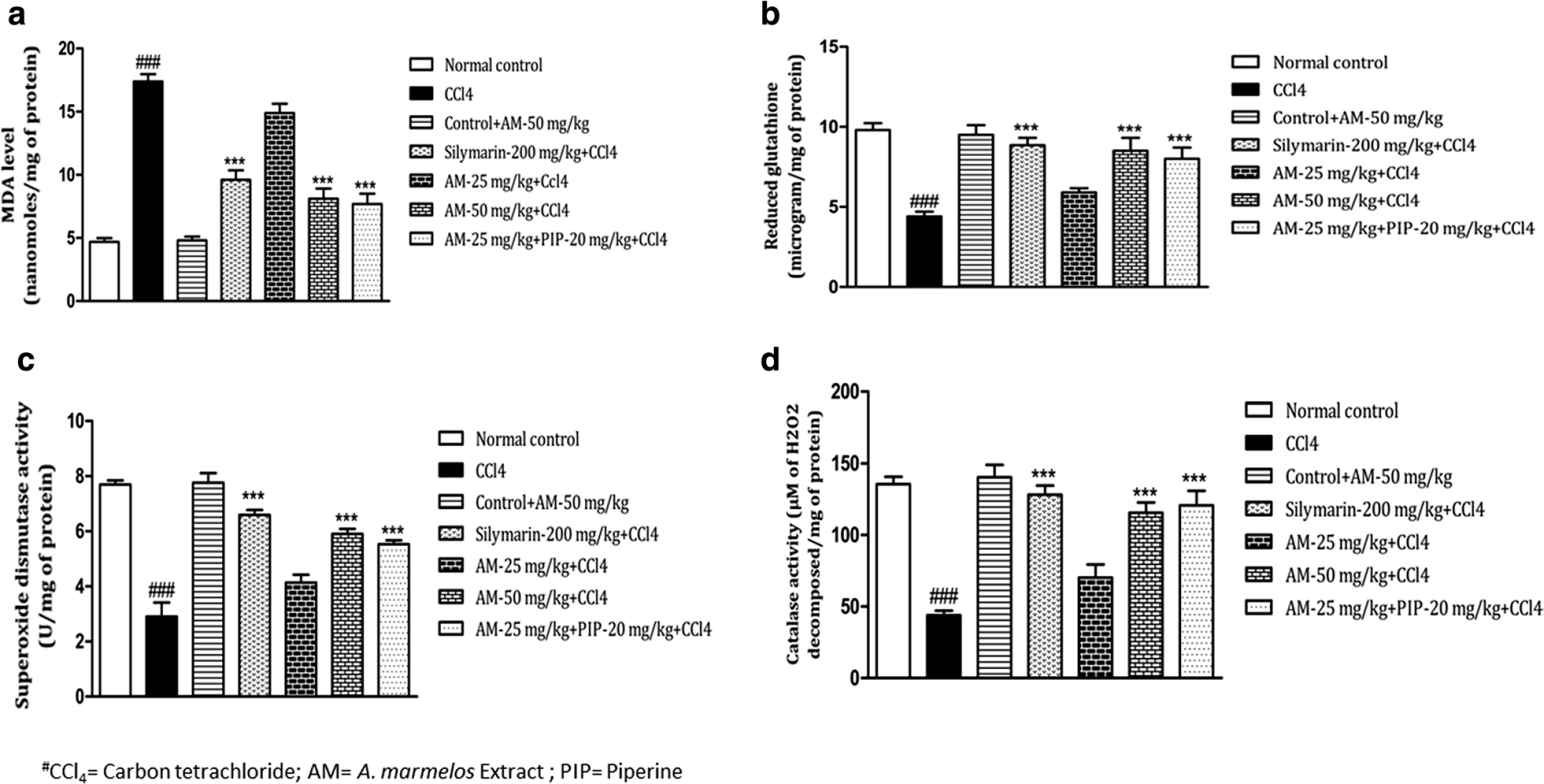
Effect of *A. marmelos* extract on hepatic **a** malondialdehyde (MDA) level; **b** reduced glutathione level; **c** superoxide dismutase (SOD) activity; **d** catalase activity in CCl_4_ induced hepatotoxicity in rats (data presented mean ± SEM; (*n* = 6); ***, ^###^*p* < 0.001; ^#^ vs Control; * vs CCl_4_)

Administration of CCl_4_ caused marked oxidative stress as indicated by significant increase in MDA level (*p *< 0.001) and decrease in reduced glutathione level (*P *< 0.001), as compared to normal control group. MDA level was significantly reduced in *A. marmelos* extract 50 mg/kg (*P *< 0.001) and *A. marmelos* extract 25 mg/kg + piperine (*p *< 0.001) treatment groups as compared to CCl_4_ group (Fig. 3a). *A. marmelos* extract 25 mg/kg group decreased MDA levels but the results were not significant as compared to CCl_4_ group. The CCl_4_ induced reduction in the reduced glutathione level was alleviated by the treatment with *A. marmelos* extract 50 mg/kg (*p *< 0.001) and *A. marmelos* extract 25 mg/kg + piperine treatment (*p *< 0.001) (Fig. 3b) as compared to CCl_4_ group. *A. marmelos* extract 25 mg/kg group increased reduced glutathione level but the results were not significant as compared to CCl_4_ group. Treatment with silymarin-200 mg/kg significantly ameliorated the CCl_4_ induced alterations in MDA (*p *< 0.001) and reduced glutathione (*p *< 0.001) levels. The results of silymarin group were comparable to *A. marmelos* extract 50 mg/kg group and *A. marmelos* extract 25 mg/kg + piperine group.

The endogenous antioxidant enzymes i.e. SOD and catalase activities were significantly lowered by the administration of CCl_4_. Alterations in the SOD activity was significantly reversed by treatment with *A. marmelos* extract 50 mg/kg (*p *< 0.001) and *A. marmelos* extract 25 mg/kg + piperine (*p *< 0.001) (Fig. 3c). Similarly, catalase activity was significantly increased with v-50 mg/kg (*p *< 0.001) treatment and *A. marmelos* extract 25 mg/kg + piperine (*p *< 0.001) treatment as compared to CCl_4_ group (Fig. 3d). The SOD and catalase activity improved with *A. marmelos* extract 25 mg/kg treatment but the difference was not significant as compared to CCl_4_ group. Silymarin treatment significantly (*p *< 0.001) ameliorated the CCl_4_ induced alterations in SOD and catalase enzymes activity. The SOD and catalase activity of *A. marmelos* extract 50 mg/kg group and *A. marmelos* extract 25 mg/kg + piperine group were comparable to silymarin group. The results of drug control group were comparable to normal control group.

### Effect of *A. marmelos* treatment on alterations in glutathione reductase, transferase, and peroxidase and glucose-6-phosphate dehydrogenase activity

**Fig. 4:**
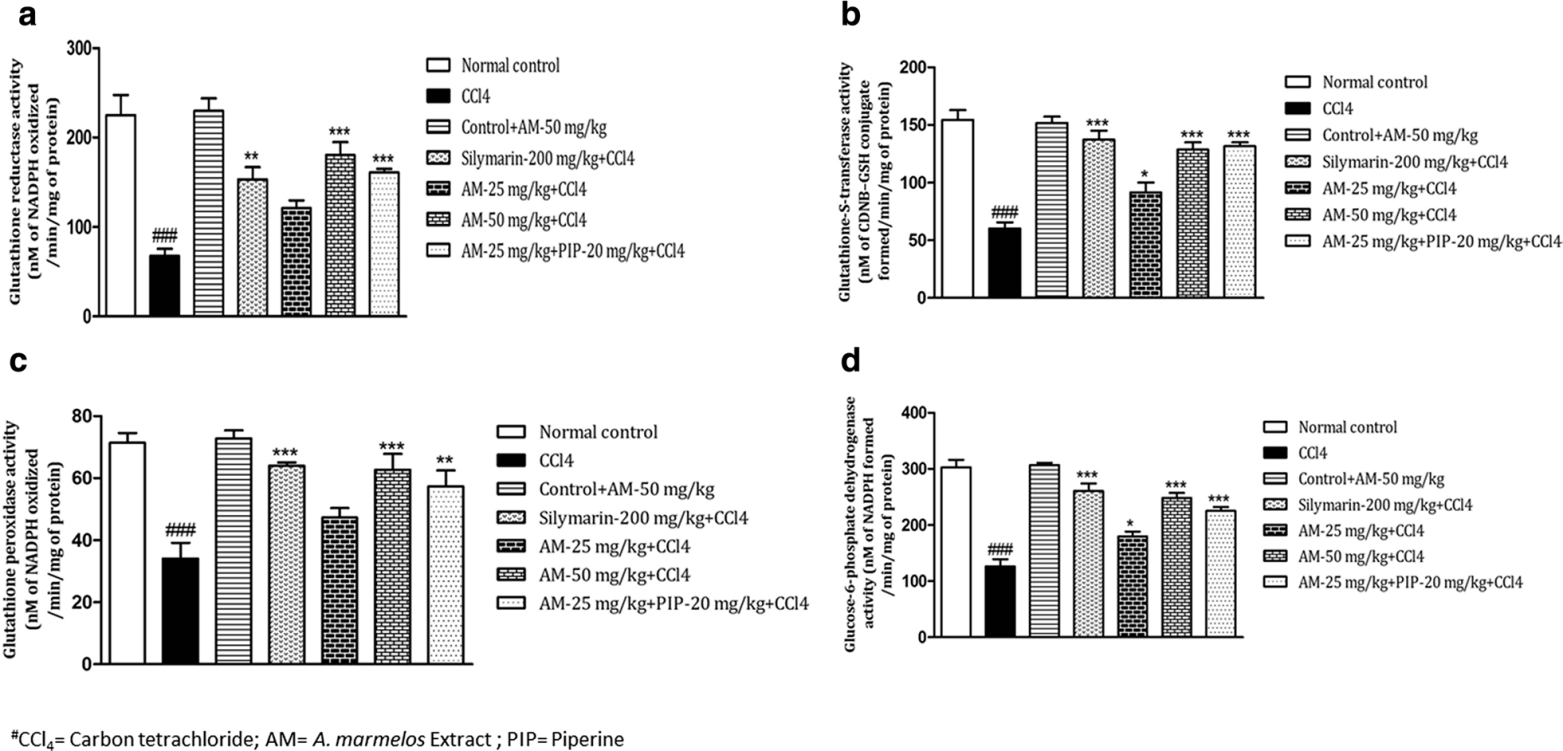
Effect of *A. marmelos* extract on **a** hepatic glutathione reductase activity; **b** hepatic glutathione S-transferase activity **c** hepatic glutathione peroxidase activity; **d** glucose-6-phosphate dehydrogenase (G6PD) in CCl_4_ induced hepatotoxicity in rats (data presented mean ± SEM; (*n* = 6); *p < 0.05; **p < 0.01; ***, ^###^*p* < 0.001; ^#^ vs Control; * vs CCl_4_)

The CCl_4_ administration caused a significant (*p *< 0.001) reduction in glutathione reductase, transferase and peroxidase and G6PD activities as compared with the normal control group. These alterations reversed significantly by treatment with *A. marmelos* extract 50 mg/kg. CCl_4_ induced reduction in glutathione reductase activity was significantly reversed by the treatment with *A. marmelos* extract 50 mg/kg (*p *< 0.001) and *A. marmelos* extract 25 mg/kg + piperine treatment (*p *< 0.001) (Fig. 4a). Glutathione transferase activity was significantly increased by *A. marmelos* 25 mg/kg (*p *< 0.05), *A. marmelos* 50 mg/kg (*p *< 0.001) and *A. marmelos* extract 25 mg/kg + piperine (*p *< 0.001) treatment as compared to CCl_4_ treated animals (Fig. 4b). Figure 4c depicts the elevation of glutathione peroxidase activity with *A. marmelos* extract 50 mg/kg (*p *< 0.001) treatment and by treatment with *A. marmelos* extract 25 mg/kg + piperine (*p *< 0.01) as compared to CCl_4_ group. G6PD activity was also increased by the treatment with *A. marmelos* 25 mg/kg (*p *< 0.05), *A. marmelos* 50 mg/kg (*p *< 0.001) and *A. marmelos* Eextract 25 mg/kg + piperine treatment (*p *< 0.001) as compared to CCl_4_ group (Fig. 4d). Silymarin 200 mg/kg significantly ameliorated CCl_4_ induced alterations in glutathione reductase (*p *< 0.01), transferase (*p *< 0.001) and peroxidase (*p *< 0.001) and G6PD (*p *< 0.001) activities. The results of *A. marmelos* extract 50 mg/kg group and *A. marmelos* extract 25 mg/kg + piperine group were comparable to silymarin group. The results of drug control group were comparable to normal control group.

### *A. marmelos* treatment and alterations in TNF-α and IL-10

**Fig. 5:**
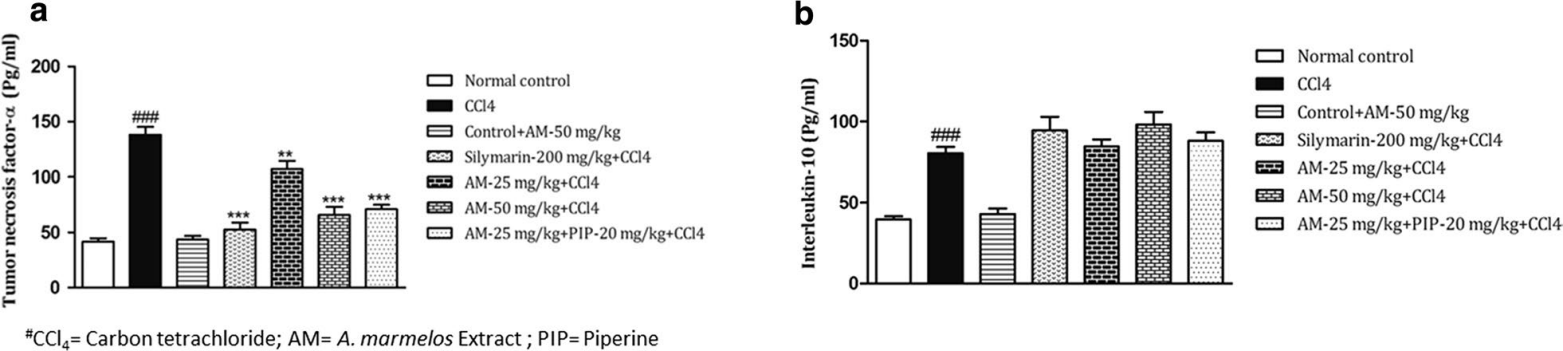
Effect of *A. marmelos* extract on **a** serum Tumor necrosis factor (TNF-α) **b** serum Interleukin (IL-10) in CCl_4_ induced hepatotoxicity in rats (data presented mean ± SEM; (*n* = 6); **p < 0.01; ***, ^###^*p* < 0.001; ^#^ vs Control; * vs CCl_4_)

Pro-inflammatory cytokine (TNF-α) was significantly increased (*p *< 0.001) in the serum of CCl_4_ administered rats as compared to normal control group. However, treatment with *A. marmelos* 25 mg/kg (*p *< 0.01), *A. marmelos* (50 mg/kg (p < 0.001) and *A. marmelos* 25 mg/kg + piperine (*p *< 0.001) significantly prevented the elevation of serum TNF-α level (Fig. 5a). IL-10 was found to be significantly higher in CCl_4_ administered group as compared to normal control group. Treatment with *A. marmelos* 25 mg/kg, *A. marmelos* 50 mg/kg and *A. marmelos* extract -25 mg/kg + piperine 20 mg/kg did not produce any significant effect on serum IL-10 level as compared to CCl_4_ group (Fig. 5b). Silymarin 200 mg/kg did not show any significant effect on serum IL-10 level but it significantly reduced CCl_4_ induced elevated serum TNF-α level. The results of drug control group were comparable to normal control group.

### Histopathology

**Fig. 6:**
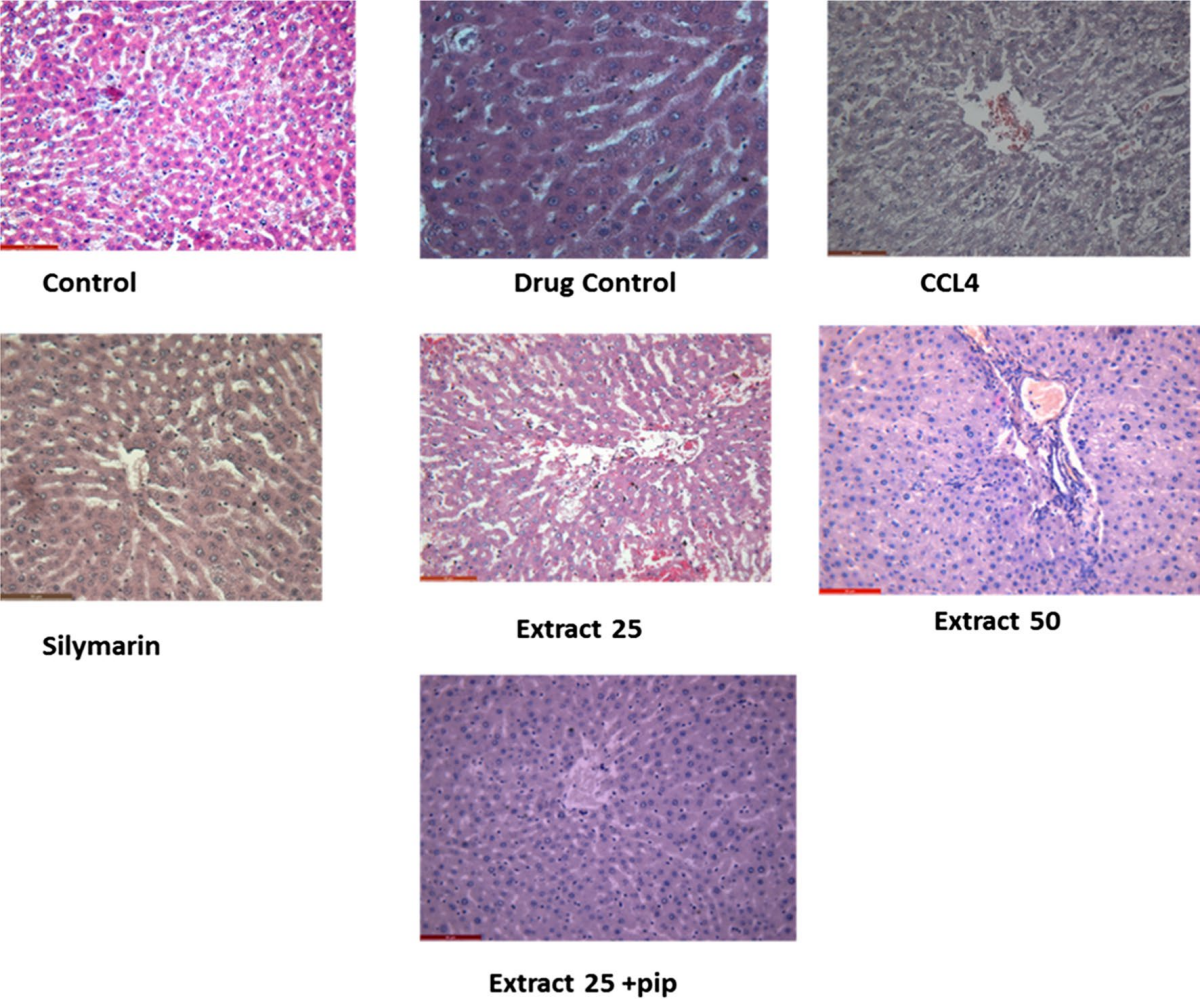
Photomicrographs depicting histological appearance in different groups

Histological analysis revealed that CCl_4_ caused marked hepatotoxicity as evident by shrinkage of central veins, hepatocellular hypertrophy and necrosis. Figure 6 shows normal architecture of hepatocytes and liver parenchyma, distinct hepatic cords and central vein in normal control group. Treatment with *A. marmelos* (50 mg/kg) and the combination with piperine reduced severity of hepatic damage as compared to CCl_4_ group. Moreover, vascular distortion and lymphocyte infiltration were also reduced in extract-50 and piperine group, which further confirms its hepatoprotective effect.

## Discussion

The published literature provides evidence that various parts of *A. marmelos* showed hepatoprotective potential. However, most of the available reports are of fruits. Moreover, either the reported dose of *A. marmelos* leaves extract was very high [39] or was not so effective at low doses for the desired hepatoprotection [9]. Additionally, the reported literature showed no evidence of relationship between the dose selections of the drug extract based on the reported LD_50_ values. The current study has covered both these gaps in the literature by demonstrating the hepatoprotective potential of standardized *A. marmelos* leaves extract in a dose dependent manner utilizing the LD_50_ data reported and providing evidence that the addition of piperine to the leaves extract of A. marmelos aids in achieving the desired hepato-protection at lower doses.

Hepatocytes are the main component that regulates various metabolic activities of liver. Distortion of this organ will result in disorder of body metabolism [40, 41]. An accidental over dosage administration of CCl_4_ can result in hepatic damage. The development of CCl_4_-induced hepatotoxicity seems to depend partly on the existence of free radicals and oxidative processes [42, 43]. For that reason, it is hypothesized that extracts/compounds possessing free radical scavenging and/or antioxidant activities could also demonstrate hepatoprotective activity against the CCl_4_ toxic effect. This is supported by claim that the combination of hepatoprotective effect and antioxidant activity synergistically prevents the process of initiation and progress of hepatocellular damage [44]. In the present study the phytochemical standardization and antioxidant potential of *A. marmelos* leaves extract was carried out followed by the evaluation of hepatoprotective potential and the augmentation of the hepatoprotective activity by co-administration of piperine. Our results demonstrated *A. marmelos* extract has the ability to scavenge free radicals and to exert antioxidant activity, using the DPPH assay, which is in agreement with literature [45]. *A. marmelos* leaves extract exhibited concentration dependent antioxidant potential. The IC_50_ of the extract was observed at 160.9 μg/ml in DPPH assay and 134.5 μg/ml in ABTS assay. The IC_50_ in FRAP assay was observed at 424.5 μg/ml which is significantly higher than the other two assay. Literature reports that many phenolic compounds having overlapping spectra may react with DPPH and ABTS which interfere with the final results [46]. Moreover, the inflammatory processes activated by CCl_4_ are intimately involved in the chemical-induced hepatotoxic processes [47]. The inflammatory processes are thought to be responsible for producing various mediators, which are involved in the production of reactive oxygen species (ROS) and nitric oxide (NO) that can affect liver damage or repair. Therefore, it is also possible to postulate that extracts/compounds possessing anti-inflammatory activity might also exhibit hepatoprotective activity. The results of the preliminary phytochemical screening of *A. marmelos* demonstrated the presence of high content of phenolic compounds in accordance with the literature reports. Furthermore, estimation of rutin in *A. marmelos* extract was performed by using HPLC. The results revealed the presence of rutin in good amounts in the hydro-alcoholic extract of *A. marmelos* leaves. Literature reports that rutin has potent hepatoprotective and anti-oxidant effects and can be used as an alternative treatment for liver diseases [48, 49]. Taking all these reports into consideration; it is plausible to suggest that the hepatoprotective activity of *A. marmelos,* was partly correlated to the synergistic effect of phenolic compounds.

CCl_4_ is a well-known hepatotoxin and is widely used experimental model of hepatic injury. ALT, AST and ALP are the cellular enzymes, which increase during hepatic injury due to the impaired transport function of hepatocytes [50]. High level of AST in the serum indicates cellular injury and disturbance in transport function of cell membrane in the liver [44]. In the current study, we found that serum ALT, AST, ALP and LDH level increased after CCl_4_ administration, which indicates hepatocellular injury. Pretreatment with *A. marmelos* and silymarin as well as combination with piperine dose dependently reduced elevated serum enzymes by maintaining integrity of hepatocellular membrane.

Bilirubin is a product formed from the breakdown of red blood cells within the reticuloendothelial system. Elevated level of bilirubin indicates impaired bilirubin transport, increased hemolysis or decreased conjugation with glucuronic acid [51]. Bilirubin is an indicator to assess the normal functioning of the liver [52]. In our study, bilirubin level was found elevated in the CCl_4_ treated group, which indicates the abnormal functioning of the liver. However, high dose of *A. marmelos* extract and concomitant piperine and low dose of *A. marmelos* extract treatment significantly reduced the elevated level of bilirubin in CCl_4_ treated group. The above results indicate that *A. marmelos* extract treatment and combined treatment of *A. marmelos* extract with piperine exerts hepatoprotective effect against CCl_4_ induced liver toxicity. The results were also comparable with silymarin group. The damage of liver cell plasma membrane leads to release of, a variety of enzymes normally located in the cytosol into the blood stream. Their estimation in the serum is a useful quantitative marker of the extent and type of hepato cellular damage [53, 54]. In the present study, the hydro-alcoholic extract of leaves of *A. marmelos* showed a potential dose dependent in vivo hepatoprotective activity as it is evident by the elevation of the reduced levels of liver cytosolic SOD, catalase, and peroxidase activity. These antioxidant enzymes are involved in the reduction of reactive oxygen species (ROS) and peroxides produced in the living organism thereby playing a vital role in maintaining the redox balance. Results revealed that the hydro-alcoholic extract has a potential to help in cellular defense mechanisms by preventing cell membrane oxidation as indicated by the restoration of the SOD activity toward normal value [55]. *A. marmelos* extract and silymarin can also play a vital role in scavenging hydrogen peroxide, as indicated by an increase in the catalase activity with respect to CCl_4_ treatment group. Similarly, an increase in the peroxidase activity indicates that the *A. marmelos* extract also helps in the restoration of vital molecules such as NAD, cytochrome, and glutathione [55].

In the current study, we found elevated level of MDA content in the liver of CCl_4_ intoxicated rats, indicating the presence of CCl_4_ evoked oxidative stress. Previous studies have shown the free radical scavenging property of *A. marmelos* extract due to presence of high phenolic components in the *A. marmelos* extract [56, 57]. Our results demonstrate that pre-treatment with *A. marmelos* significantly reduced MDA content indicating a decrease of oxidative damage. Our findings are in parallel with previous reported studies, which indicated potent antioxidant potential of *A. marmelos* [45, 56, 57]. Further, the HPLC results of the extract revealed the presence of rutin in high amounts thereby supporting the hepatoprotective results.

Inflammatory cells are recruited and activated due to direct oxidative stress, which can intensify liver damage by the release of several inflammatory mediators, including cytokines [58]. TNF-α is a pleiotropic cytokine with numerous immunologic and metabolic actions. The TNF-α activity is increased in liver cirrhosis and generally thought to be associated with several known cirrhosis related complications such as hyperdynamic circulation, susceptibility to infection, and hepatic encephalopathy [59]. Experimental data from animal models and clinical data from patients suggest that inflammation-associated cytokines including pro-inflammatory cytokines such as TNF-α and Transforming growth factor-β (TGF-β), and anti-inflammatory cytokines such as IL-10, are involved in the development of liver injury. The effects of IL-10 have been observed in viral or autoimmune hepatitis, alcoholic liver disease, and animal models [60]. Our results demonstrate that the increased TNF-α level due to CCl_4_ induced liver injury were effectively reduced by the pretreatment with *A. marmelos* and silymarin. However, IL–10 levels were not reduced which indicated that the mechanism of hepatoprotection showed by *A.* marmelos was not through decreasing the levels of anti-inflammatory cytokines and it remains elusive. To ascertain the mechanism, further studies are warranted before we could conclude on the exact mechanism(s) involved in the hepatoprotective activity of the *A. marmelos*.

## Conclusion

The results of this study demonstrate that *A. marmelos* leaf extract possess hepatoprotective properties. These properties could be linked to the presence of phenolic compounds as confirmed by the phytochemical screening and to their antioxidant properties. Literature is also supportive of the fact that phenolic compounds possess hepatoprotective and antioxidant properties [61, 62]. In addition, rutin was the major phenolic compound available in this plant as was evident from HPLC study. Hence it could be postulated that the hepatoprotective potential of the *A. marmelos* leaves could be attributed to rutin. Moreover, *A. marmelos* extract significantly reduced the elevated TNF-α level thus indicating the possibility of reducing the pro-inflammatory cytokines. Further, the results of this work showed a comparable hepatoprotective effect of *A. marmelos* to that of silymarin. The hepatoprotective activity of *A. marmelos* leaves extract was augmented when piperine was co-administered. The augmentation of hepatoprotective effect was possibly due to the bio-enhancing properties of piperine or by the synergistic effect of phyto-constituents available in *A. marmelos* and piperine. All our experimental results revealed that *A. marmelos* leaves extract could be formulated in combination of piperine for enhanced liver protection against several liver toxicants such as CCl_4_. Therefore, the results of the present study are in accordance with the previously reported results revealing the hepatoprotective potential of the plant. Majority of the published literature provides evidence regarding the hepatoprotective potential of fruits of *A. marmelos*. Furthermore, the published studies reporting the hepatoprotective potential of leaves were on crude leaf powder and on very high doses without any reference to LD_50_ data for the plant extract [9, 39]. However the current study demonstrated the hepatoprotective potential of standardized *A. marmelos* leaves extract in a dose dependent manner utilizing the LD_50_ data reported. Moreover, our study provides evidence that the addition of piperine to the leaves extract of *A. marmelos* aids in achieving the desired hepatoprotection at lower doses. Additionally, the considerable amount of rutin might be responsible for the enhanced hepatoprotective activity of *A. marmelos*, however, the mechanism of action still needs to be studied.

In summary, the results strongly indicated that *A. marmelos* and *A. marmelos *+ piperine exhibited considerable hepatoprotection, which is comparable to that of silymarin. This study provides vital evidence about the hepatoprotective potential of *A. marmelos* prospecting this for developing potential hepatoprotective therapeutics.

## References

[1] Agarwal SS, Tamrakar BP, Paridhavi M (2005). Clinically useful heabal drugs.

[2] Kirtikar KR, Basu BD (1993). Indian medicinal plants.

[3] Balakumar S, Rajan S, Thirunalasundari T, Jeeva S (2011). Antifungal activity of *Aegle marmelos* (L.) Correa (Rutaceae) leaf extract on dermatophytes. Asian Pac J Trop Biomed.

[4] Das SK, Roy C (2012). The protective role of *Aegle marmelos* on aspirin–induced gastro-duodenal ulceration in albino rat model: a possible involvement of antioxidants. Saudi J Gastroenterol.

[5] Benni JM, Jayanthi MK, Suresha RN (2011). Evaluation of the anti-inflammatory activity of *Aegle marmelos* (Bilwa) root. Indian J Pharmacol.

[6] Sabu MC, Kuttan R (2004). Antidiabetic activity of *Aegle marmelos* and its relationship with its antioxidant properties. Indian J Physiol Pharmacol.

[7] Singh S, Singh SK, Srivastava S, Singh P, Trivedi M, Shanker P, Dixit RK, Rana RS (2013). Experimental evaluation of diuretic activity of *Aegle marmelos* in rats. Int J Pharm Biol Sci.

[8] Agrawal SS, Kumar A, Gullaiya S, Dubey V, Nagar A, Tiwari P, Dhar P, Singh V (2012). Antifertility activity of methanolic bark extract of *Aegle marmelos* (L.) in male wistar rats. DARU J Pharm Sci.

[9] Singanan V, Singanan M, Begum H (2007). The hepatoprotective effect of bael leaves (*Aegle marmelos*) in alcohol induced liver injury in albino rats. Int J Sci Tech.

[10] Baliga MS, Thilakchand KR, Rai MP, Rao S, Venkatesh P (2013). *Aegle marmelos* (L.) Correa (Bael) and its phytochemicals in the treatment and prevention of cancer. Integr Cancer Ther.

[11] Tasleem F, Azhar I, Ali SN, Perveen S, Mahmood ZA (2014). Analgesic and anti-inflammatory activities of *Piper nigrum* L.. Asian Pac J Trop Med.

[12] Mehmood MH, Gilani AH (2010). Pharmacological basis for the medicinal use of black pepper and piperine in gastrointestinal disorders. J Med Food.

[13] Koul IB, Kapil A (1993). Evaluation of the liver protective potential of piperine, an active principle of black and long peppers. Planta Med.

[14] Avasarala S, Yang L, Sun Y, Leung AWC, Chan WY, Cheung WT, Lee SST (2006). A temporal study on the histopathological, biochemical and molecular responses of CCl_4_-induced hepatotoxicity in Cyp2e1-null mice. Toxicology.

[15] Recknagel RO, Lombardi B, Schotz MC (1960). A new insight into pathogenesis of carbon tetrachloride fat infiltration. Proc Soc Exp Biol Med.

[16] Terao J, Asano I, Matsushita S (1984). High-performance liquid chromatographic determination of phospholipid peroxidation products of rat liver after carbon tetrachloride administration. Arch Biochem Biophys.

[17] Harbrone JB (1973). Phytochemicals methods.

[18] Kokate CK (1986). Practical pharmacognosy.

[19] Zu Y, Li C, Fu Y, Zhao C (2006). Simultaneous determination of catechin, rutin, quercetin kaempferol and isorhamnetin in the extract of sea buckthorn (*Hippophae rhamnoides* L.) leaves by RP-HPLC with DAD. J Pharm Biomed Anal.

[20] Landim LP, Feitoza GS, Costa JGM (2013). Development and validation of a HPLC method for the quantification of three flavonoids in a crude extract of *Dimorphandra gardneriana*. Rev bras farmacogn.

[21] Molyneux P (2004). The use of the stable free radical diphenylpicrylhydrazyl (DPPH) for estimating antioxidant activity. Songklanakarin J. Sci. Technol.

[22] Miller NJ, Rice-Evans CA (1997). Factors influencing the antioxidant activity determined by the ABTS· + radical cation assay. Free Radic Res.

[23] Gil MI, Tomás-Barberán FA, Hess-Pierce B, Kader AA (2002). Antioxidant capacities, phenolic compounds, carotenoids, and vitamin C contents of nectarine, peach, and plum cultivars from California. J Agric Food Chem.

[24] Adewale OB, Adekeye AO, Akintayo CO, Onikanni A, Sabiu S (2014). Carbon tetrachloride (CCl_4_)-induced hepatic damage in experimental Sprague Dawley rats: antioxidant potential of *Xylopia aethiopica*. J Phytopharmacol.

[25] Veerappan A, Miyazaki S, Kadarkaraisamy M, Ranganathan D (2007). Acute and subacute toxicity studies of *Aegle marmelos* Corr., an Indian medicinal plant. Phytomed.

[26] Rasool M, Iqbal J, Malik A, Ramzan HS, Qureshi MS, Asif M, Qazi MH, Kamal MA, Chaudhary AG, Al-Qahtani MH, Gan SH (2014). Hepatoprotective effects of *Silybum marianum* (Silymarin) and *Glycyrrhiza glabra* (Glycyrrhizin) in combination: a possible synergy. Evid Based Comp Altern Med.

[27] Hiwale AR, Dhuley JN, Naik SR (2002). Effect of co-administration of piperine on pharmacokinetics of β-lactam antibiotics in rats. Indian J Exp Biol.

[28] Lowry OH, Rosebrough NJ, Farr AL, Randall RJ (1951). Protein measurement with the Folin phenol reagent. J Biol Chem.

[29] Ohkawa H, Ohishi N, Yagi K (1979). Assay for lipid peroxides in animal tissues by thiobarbituric acid reaction. Anal Biochem.

[30] Ellman GL (1959). Tissue sulfhydryl groups. Arch Biochem Biophys.

[31] Robak J, Gryglewski RJ (1988). Flavonoids are scavengers of superoxide anions. Biochem Pharmacol.

[32] Aebi H, Bergmeyer HU (1974). Catalase. Methods of enzymatic analysis.

[33] Carlberg IN, Mannervik BE (1975). Purification and characterization of the flavoenzyme glutathione reductase from rat liver. J Biol Chem.

[34] Habig WH, Pabst MJ, Jakoby WB (1974). Glutathione S-transferases the first enzymatic step in mercapturic acid formation. J Biol Chem.

[35] Athar MO, Iqbal MO (1998). Ferric nitrilotriacetate promotes N-diethylnitrosamine-induced renal tumorigenesis in the rat: implications for the involvement of oxidative stress. Carcinogenesis.

[36] Zaheer N, Tewari KK, Krishnan PS (1965). Exposure and solubilization of hepatic mitochondrial shunt dehydrogenases. Arch Biochem Biophys.

[37] Siddique NA, Mujeeb M, Najmi AK, Akram M (2010). Evaluation of antioxidant activity, quantitative estimation of phenols and flavonoids in different parts of *Aegle marmelos*. Afr J Plant Sci.

[38] Nair CJ, Ahamad S, Khan W, Anjum V, Mathur R (2017). Development and validation of high-performance thin-layer chromatography method for simultaneous determination of polyphenolic compounds in medicinal plants. Pharmacognosy Res.

[39] Jayachandra K, Sivaraman T (2011). Hepatoprotective effect of *Aegle Marmelos* (L.) Corr. Leaf powder (Crude) against carbon tetrachloride-induced hepatic damage in albino rats. J Pharma Sci Res.

[40] Shaker E, Mahmoud H, Silymarin Mnaa S (2010). Silymarin, the antioxidant component and *Silybum marianum* extracts prevent liver damage. Food Chem Toxicol.

[41] Zakaria ZA, Rofiee MS, Somchit MN, Zuraini A, Sulaiman MR, Teh LK, Salleh MZ, Long K (2011). Hepatoprotective activity of dried-and fermented-processed virgin coconut oil. Evid Based Complement Altern Med.

[42] Hazai E, Vereczkey L, Monostory K (2002). Reduction of toxic metabolite formation of acetaminophen. Biochem Biophys Res Commun.

[43] Rajkapoor B, Venugopal Y, Anbu J, Harikrishnan N, Gobinath M, Ravichandran V (2008). Protective effect of *Phyllanthus polyphyllus* on acetaminophen induced hepatotoxicity in rats. Pak J Pharm Sci.

[44] Gupta AK, Chitme H, Dass SK, Misra N (2006). Hepatoprotective activity of *Rauwolfia serpentina* rhizome in paracetamol intoxicated rats. J Pharmacol Toxicol.

[45] Reddy VP, Urooj A (2013). Antioxidant properties and stability of *Aegle marmelos* leaves extracts. J Food Sci Tech.

[46] Prior RL, Wu X, Schaich K (2005). Standardized methods for the determination of antioxidant capacity and phenolics in foods and dietary supplements. J Agric Food Chem.

[47] Luster MI, Simeonova PP, Gallucci RM, Matheson JM, Yucesoy B (2000). Immunotoxicology: role of inflammation in chemical-induced hepatotoxicity. Int J Immunopharmacol.

[48] Domitrović R, Jakovac H, Marchesi VV, Vladimir-Knežević S, Cvijanović O, Tadić Ž, Romić Ž, Rahelić D (2012). Differential hepatoprotective mechanisms of rutin and quercetin in CCl4-intoxicated BALB/cN mice. Acta Pharmacol Sin.

[49] Hafez MM, Al-Harbi NO, Al-Hoshani AR, Al-Hosaini KA, Al Shrari SD, Al Rejaie SS, Sayed-Ahmed MM, Al-Shabanah OA (2015). Hepato-protective effect of rutin via IL-6/STAT3 pathway in CCl 4-induced hepatotoxicity in rats. Biol Res.

[50] Giannini EG, Testa R, Savarino V (2005). Liver enzyme alteration: a guide for clinicians. Can Med Assoc J.

[51] Sasidharan S, Aravindran S, Latha LY, Vijenthi R, Saravanan D, Amutha S (2010). In vitro antioxidant activity and hepatoprotective effects of *Lentinula edodes* against paracetamol-induced hepatotoxicity. Molecules.

[52] Abirami A, Nagarani G, Siddhuraju P (2015). Hepatoprotective effect of leaf extracts from *Citrus hystrix* and *C. maxima* against paracetamol induced liver injury in rats. Food Sci Human Well.

[53] Mitra SK, Venkataranganna MV, Sundaram R, Gopumadhavan S (1998). Protective effect of HD-03, a herbal formulation, against various hepatotoxic agents in rats. J Ethnopharmacol.

[54] Mondal A, Karan SK, Singha T, Rajalingam D, Maity TK (2012). Evaluation of hepatoprotective effect of leaves of *Cassia sophera* Linn”. Evid Based Complement Altern Med.

[55] Lee S, Lee YS, Jung SH, Kang SS, Shin KH (2003). Anti-oxidant activities of fucosterol from the marine algae Pelvetia siliquosa. Arch Pharm Res.

[56] Kamalakkannan N, Prince PSM (2003). Hypoglycaemic effect of water extracts of *Aegle marmelos* fruits in streptozotocin diabetic rats. Ethnopharmacol.

[57] Kamalakkannan N, Stanely MPP (2003). Effect of *Aegle marmelos* Correa. (Bael) fruit extract on tissue antioxidants in streptozotocin diabetic rats. Indian J Exp Biol.

[58] Xin Y, Wei J, Chunhua M, Danhong Y, Jianguo Z, Zongqi C, Jian-an B (2016). Protective effects of ginsenoside Rg1 against carbon tetrachloride-induced liver injury in mice through suppression of inflammation. Phytomed.

[59] Elzefzafy WM, AboulEla A, Maabady MH, Shahin RS (2013). Role of tumor necrosis factor alpha, Ghrelin, evoked potentials in hepatic encephalopathy. Egypt J Hosp Med.

[60] Zhang LJ, Wang XZ (2006). Interleukin-10 and chronic liver disease. World J Gastroenterol.

[61] Shimoda H, Tanaka J, Kikuchi M, Fukuda T, Ito H, Hatano T, Yoshida T (2008). Walnut polyphenols prevent liver damage induced by carbon tetrachloride and D-galactosamine: hepatoprotective hydrolyzable tannins in the kernel pellicles of walnut. J Agric Food Chem.

[62] Bhoopat L, Srichairatanakool S, Kanjanapothi D, Taesotikul T, Thananchai H, Bhoopat T (2011). Hepatoprotective effects of lychee (*Litchi chinensis* Sonn.): a combination of antioxidant and antiapoptotic activities. J Ethnopharmacol.

